# Unmet Needs and Strategies to Promote Patient Engagement in the Arab World: Experts’ Opinion

**DOI:** 10.7759/cureus.56804

**Published:** 2024-03-24

**Authors:** Yehia Nabil, Anwar Eldaw, Dalia El-Shourbagy, Dima Ibrahim, Hanan Alturkistani, Mohammed Alshahrani, Mohamed Farghaly, Sawsan AlMadhi, Romy Mansour

**Affiliations:** 1 Medical Affairs, Gilead Sciences Inc., Dubai, ARE; 2 Infectious Disease/Public Health and Preventive Medicine, Ministry of Health, Riyadh, SAU; 3 Medical Oncology, Dubai Hospital, Dubai, ARE; 4 Transplant Infectious Diseases, Burjeel Medical Center, Abu Dhabi, ARE; 5 Transplant Infectious Diseases, King Abdullah Medical City, Makkah, SAU; 6 Pediatric Hematology/Oncology, Prince Sultan Military Medical City, Riyadh, SAU; 7 Family Medicine/Public Health, Dubai Medical College, Dubai, ARE; 8 Advocacy, AlignnEficient Health Consultancies, Dubai, ARE; 9 Ophthalmology, Lebanese American University Medical Center-Rizk Hospital, Beirut, LBN

**Keywords:** healthcare system, healthcare services, patient satisfaction, patient experience, patient engagement, arab countries

## Abstract

Background: The significance of patient engagement (PE) is widely acknowledged as a crucial element in fostering positive health outcomes, elevating care quality, and streamlining healthcare systems. Despite its recognized advantages, the level of patient engagement in Arab nations remains suboptimal.

Methods: A high-level assembly was convened in Dubai with 11 distinguished patient advocates from diverse Arab countries. Their collective aim was to dissect the obstacles hindering patient engagement in the Arab world and propose pragmatic strategies to surmount them. First, a series of five open-ended, comprehensive questions were posed and thoroughly deliberated upon. Second, the barriers to patient engagement within the experts’ respective communities were debated. A qualitative thematic analysis was conducted and two reports were generated by two independent researchers from the original meeting recordings.

Results: This paper highlights the importance of patient engagement in advancing healthcare and categorizes barriers to patient engagement as patient-related, provider-related, or system/government-related. The experts identified the primary gaps in patient engagement and proposed strategies to promote it, with a primary focus on motivating both patients and providers toward shared decision-making.

Conclusions: This paper amalgamates the insights and recommendations distilled from the expert gathering, juxtaposing them within the broader context of existing literature on patient engagement. Offering a comprehensive viewpoint, this article delves into the challenges and opportunities intrinsic to bolstering patient engagement in the Arab world. Moreover, it spotlights invaluable tools often overlooked within Arab countries. The practical insights provided here can serve as a roadmap for administrators and decision-makers, providing guidance to enhance patient engagement on both a national and institutional scale.

## Introduction

Patient engagement (PE) delineates the active participation of patients in decisions concerning their healthcare, demonstrating a profound ability to empower patients and significantly enhance healthcare outcomes [1]. The extent and facets of PE exhibit variability contingent upon factors such as the setting, context, patient attributes, and disease characteristics [2]. Within the realm of patient empowerment, various components align with PE, encompassing the act of posing questions, collaborating in decision-making processes, granting patients autonomy over their care, and eliciting patient feedback [1,2].

The nature of PE is contingent upon a myriad of factors, including patients' comprehension of their condition, their rapport and reliance on healthcare providers, and their access to care [1]. The unique attributes of individual patients dictate the extent to which they should participate and engage in decisions regarding their healthcare [3]. Consequently, patients can be broadly categorized based on their medical expertise, capacity for informed decision-making, and whether their primary ailment is chronic or acute [3]. It is imperative to discern between medical knowledge and the general level of education. For instance, a patient holding a Ph.D. in literature might exhibit less inclination to engage in their healthcare decisions compared to a science student.

Patients with higher educational attainment, decision-making capability, and chronic health issues have a deeper and more involved level of engagement than those with lower educational levels, impaired decision-making capacities, or acute health problems [4]. These predictors of PE can amalgamate into an overall PE score that positively correlates with the recommended level of engagement for optimal health outcomes and patient contentment [4]. While acknowledging this correlation aids providers in tailoring engagement levels to individual patient needs and expectations, the issues in engaging patients with transient, acute problems lack substantial literature.

At its essence, PE empowers patients by bestowing a sense of authority over their healthcare. Yet, the value of PE transcends patient outcomes and satisfaction, influencing research, protocols, and overall care quality [1]. Educating patients about their condition and management plan nurtures this sense of authority and accountability. It amplifies shared decision-making processes and encourages the regular solicitation of feedback concerning concerns, adherence, and potential side effects [4]. Achieving adequate levels of PE mandates significant time investment from both patients and providers, coupled with healthcare system optimization and strategic utilization of appropriate media channels to target diverse community segments [5].

While the literature thoroughly establishes the importance of PE, a dearth of information exists regarding PE in Arab countries. In this expert opinion and narrative review, we amalgamate insights and recommendations from an expert meeting within the broader spectrum of existing literature on PE, offering a comprehensive outlook on the challenges and prospects associated with promoting PE in the Arab world.

This article was previously posted to the ResearchSquare preprint platform on January 3, 2024. 

## Materials and methods

Setting and participants

A high-level meeting was convened in Dubai, United Arab Emirates (UAE) bringing together 11 prominent advocates for PE. These experts hailed from various Arab countries, including Saudi Arabia, UAE, Egypt, and Lebanon. All the experts possessed extensive experience in the field of PE, public health, and patient care, ranging from medical doctors, healthcare consultants, and officials from the Ministry of Health to nurses, motivational speakers, and cancer survivors. This diverse panel ensured a rich exchange of perspectives and insights during the meeting. The authors of this paper comprise the experts who actively participated in the meeting.

Approach

In the first part of the meeting, a set of five open-ended comprehensive questions were prepared to guide the discussion (Table 1). In the second part, Experts were asked to list the barriers against PE in their communities and suggest ways to overcome them. Experts were divided into two groups allowing for more suggestions to be proposed. The meeting was recorded.

**Table 1:** Questions that guided the group discussion

Questions that guided the group discussion
1. How do you see the value of patient engagement/inclusions?
2. What could be the tools to identify patient engagement models?
3. How can we motivate the patients to be engaged?
4. How can we foster the medical community towards more patient involvement?
5. What could be the instrumental solutions/ideas to leverage patient engagement? (Define, Enumerate, and Describe How)

Analysis

The recordings from both groups and the common discussion were transcribed. Despite the structured approach used during the meeting, a thematic analysis was conducted for the transcript in order to remove redundancies and group-related ideas. Two reports were then generated by two independent researchers. All ideas deemed relevant to the topic were extracted and reported in the present Expert Opinion paper. Various suggestions were supported where possible with literature.

## Results

Expert insights

The Value of PE

PE plays a critical role in advancing healthcare on various levels [6]. Encouraging patients to engage in their healthcare is essential for achieving patient-centered care, improving health outcomes, and fostering trust between patients and healthcare providers [6]. PE also benefits physicians, healthcare systems, and societies [7]. It can result in improved healthcare outcomes, decreased rates of preventable medical errors, efficient allocation of resources, and higher cost-effectiveness for healthcare systems and communities [8]. For healthcare providers, PE can lead to decreased medical errors and misdiagnoses, improved compliance, trust, and enhanced medical research outcomes [9]. Patients who engage in their healthcare experience a reduction in the duration and severity of symptoms with an associated reduction in treatment costs and re-admission rates. They feel empowered and responsible for their health [6]. Overall, PE is a vital aspect of healthcare delivery that can lead to better health outcomes for patients, healthcare providers, and healthcare systems [8].

PE is an old concept, and its value is well-established. However, to date, multiple barriers impede the attainment of proper PE in healthcare [2]. These can be classified into three categories: patient-related, provider-related, and system/government-related.

Patient-Related Barriers to PE

Multiple barriers discourage patients from engaging with their healthcare plan. They include the lack of awareness of the importance of PE and the fear and anxiety associated with more knowledge or a new diagnosis, patients' fear of stigmatization, the need for empowerment, and the lack of sufficient information about their health condition and management options [2,10]. Patients are rarely familiar with their rights and the idea that they are responsible for their health [7].

Patients with limited health literacy may have difficulty understanding medical jargon or treatment options [2]. Moreover, language and cultural barriers may create communication difficulties for patients who do not speak the same language as their healthcare providers or come from different cultural backgrounds. Finally, patients who do not trust their healthcare providers or the healthcare system may also be reluctant to engage fully in their healthcare [6].

Provider and System-Related Barriers to PE

Time constraints in busy clinical settings are one of the main obstacles because a limited time with patients makes it challenging to engage patients fully [11]. Language or cultural barriers prevent effective communication, especially when healthcare providers lack training in PE techniques, such as shared decision-making, motivational interviewing, or patient education [12]. Language barriers or communication gaps between healthcare providers and patients can impede effective engagement. The Arab world has diverse populations with different languages and dialects, making communication challenging in healthcare settings. Finally, providers may not be incentivized to engage patients fully in their care, particularly in healthcare models where providers are paid based on the number of services provided rather than patient outcomes [13].

Organizational barriers include limited resources, staff, technology, and funding. Inadequate technology or tools, including patient portals, telehealth, or remote monitoring devices in developing countries, promote the belief that a patient's opinion is unimportant [14]. Fragmented care makes it difficult to coordinate care and engage patients fully. Limited patient access to healthcare services, particularly for marginalized or underserved populations, can further complicate PE efforts, especially in healthcare systems prioritizing their needs over those of patients [2]. Inadequate patient education, coupled with complex healthcare policies that are difficult for patients to understand, can make it challenging for patients to engage fully in their healthcare [15]. Finally, the lack of accountability for engaging patients in their care discourages efforts to improve PE [16].

Further obstacles not recognized during the meeting include challenges faced by patients with demanding schedules or conflicting priorities, potentially impeding their attendance at appointments or involvement in self-care practices. The compounded issue arises with restricted access to healthcare services and financial barriers, particularly affecting preventive care and instances necessitating multiple follow-up appointments [12]. Attitudes and perceptions, such as a lack of regard for patient autonomy or the assumption that patients are disinterested or incapable of actively engaging in their healthcare, also pose hurdles to fostering PE [15,17].

Strategies to Promote PE

Strategies to promote PE can be categorized by stakeholders into ways to motivate patients to be engaged, ways to motivate providers to involve patients, and organizational methods that can enable and facilitate PE. A summary of the main barriers and strategies for PE is detailed in Table 2. 

**Table 2:** Summary of main gaps and effective strategies in patient engagement PE Patient engagement

Patient Journey	Stakeholder	Patient Engagement	
		Challenge/Barrier	Strategy/Solution
Awareness	Authorities	Lack of Health literacy	Include preventive medicine and essential healthcare awareness in school education
	Authorities	Lack of comprehensive programs	Exhaustive National Awareness programs with a focus on preventable, most prevalent local and chronic diseases
	Authorities	Misleading information by non-experts	Leveraging technology to provide correct knowledge. Set guidelines to prevent non-experts from teaching medical information.
Diagnosis	Physicians	Time constraints	Set a limited number of patients per clinic
	Patients	Fear and anxiety	Train Health care providers on proper communication and listening skills
	Government	Limited access to healthcare	Integrated healthcare pathways, telehealth, and clear referral systems
		Fragmented care	Multidisciplinary teams and unified patient records
		Limited resources	Increase the budget to cover the procurement of needed tests and material
Treatment	Authorities	Inadequate technology	introduce patient portals and telehealth systems
	Physicians	Lack of accountability	Solicit patients' feedback through an anonymous reporting system and patient satisfaction surveys.
	Patients	Lack of understanding of complex healthcare policies	Have trained medical staff in call centers to help in patient navigation and basic inquiries
Throughout patient journey	All stakeholders	Unaware of the value of PE	Educating all stakeholders about the value of PE via national, local, and institutional campaigns and awareness programs as well as via social media.
	Patients	Language and cultural barriers	Provide multilingual material and resources.
	Physicians	Language and cultural barriers	Professional development opportunities for providers

## Discussion

The results provided in this qualitative thematic analysis are important in informing administrators about strategies that can be employed to enhance PE. Many of the ideas and suggestions presented during the meeting are largely supported in the literature [2,7].

In order to motivate patients to engage with their care plan, clear communication is key. Healthcare providers should encourage patients to ask questions and express their concerns to improve their understanding of their health and treatment [15]. Patients need to have educational materials customized to meet their interests and learning preferences, with multilingual material catering to patients from different cultural backgrounds [2].

Patients should be given opportunities to express their preferences and participate in shared decision-making [7]. Personalized care helps meet each patient's unique needs and preferences regarding the form and level of engagement. It is also essential to explain to patients the value and necessity of their engagement in a way that makes them see it as a need and not an option [15]. Finally, having trained healthcare workers in call centers to provide quick answers improve access to care and patients' navigation, and employing ways to empower patients to communicate with their providers, such as having a caretaker who knows the patient's medical history and has time to listen, and communicating with patients as equals without underestimating their understanding is crucial to motivate patients to be involved [7].

Several strategies can be employed to solve the attitudinal and belief problem and foster a medical community that prioritizes patient involvement [15]. First, healthcare providers should practice building trust with patients and communicating information with empathy, using techniques such as the sandwich method, highlighting positive aspects, and maintaining realistic expectations [6]. Additionally, providers should receive training and education on the importance of PE, implementing key performance indicators that promote a patient-centered culture, active listening, and encouraging feedback through surveys and focus groups [15].

Technology such as electronic health records, online portals, and mobile apps can facilitate patient involvement just as involving patients in quality improvement initiatives by inviting them to participate in focus groups, committees, and advisory councils can ensure that quality improvement projects are aligned with patient needs and preferences [14]. Regular patient experience and satisfaction surveys can also provide valuable insights [16]. Finally, improving financial coverage for all healthcare services can empower all patients and significantly improve their engagement [18].

To achieve PE, stakeholders can employ various tactics depending on the stage of the patient's journey. During the awareness stage, integrating primary preventive medicine education into school curricula can improve community awareness and encourage people to participate in their healthcare decisions. Providing clear and accessible information about a patient's condition and medications can empower patients to engage more effectively [7,15]. During the diagnosis stage, integrating healthcare pathways, training healthcare providers on communication skills, and involving patients in decision-making can enhance the quality and outcome of healthcare [11]. In the treatment stage, leveraging technology and social media to connect with patients, providing patient support programs, and involving patients' representatives in decision-making, quality improvement projects, and research can improve continuity of care and outcomes [11].

Cultural differences play a significant role [19]. In some Arab societies, there might be a traditional hierarchical view of the doctor-patient relationship, where the doctor's authority is unquestioned. This can discourage patients from actively participating in their healthcare decisions. Limited policies or initiatives that promote patient-centered care or encourage PE might contribute to lower levels of active involvement in healthcare decisions. The stigma associated with certain health conditions or seeking healthcare services might discourage individuals from actively engaging with healthcare providers.

The strengths of this study lie in its comprehensive approach to gathering insights and recommendations on PE in the Arab world. By convening a high-level meeting of prominent advocates with extensive experience in PE, public health, and patient care, the study ensured a diverse range of perspectives from various Arab countries, including Saudi Arabia, UAE, Egypt, and Lebanon. This amalgamation of expertise provided a rich source of information that supplements the existing literature, filling the gap regarding PE in Arab countries. Moreover, the inclusion of a cancer survivor patient and motivational speaker from Egypt among the experts ensured that the patient's perspective was not overlooked, enriching the discussions with firsthand experiences and insights.

However, limitations exist within the study framework. Firstly, the qualitative nature of the thematic analysis may limit generalizability, as findings may not be universally applicable across all Arab countries due to cultural, social, and healthcare system differences. Moreover, having experts only from four Arab countries could limit its generalizability across all other Arab countries. Additionally, the study's reliance on expert opinions could introduce biases, potentially overlooking the perspectives of patients themselves or other stakeholders within the healthcare system. Furthermore, the meeting's exclusive focus on prominent advocates for PE may result in a lack of representation of diverse voices, such as those from marginalized communities or patients with different socioeconomic backgrounds. Despite these limitations, the study provides valuable insights and recommendations for administrators seeking to enhance PE in the Arab world, laying a foundation for future research and initiatives in this important area.

## Conclusions

PE is a collective responsibility that requires the active participation of patients, healthcare providers, the healthcare system, and government agencies. A patient-centered approach that prioritizes the needs and preferences of patients is critical for supporting patients in their healthcare journey. Educating stakeholders about the value and benefits of PE is an important first step in achieving this goal. By prioritizing PE and providing necessary resources and support, a more effective healthcare system can be created.

Addressing these disparities in PE in the Arab world involves implementing culturally sensitive approaches, improving health literacy, enhancing communication between patients and healthcare providers, investing in healthcare infrastructure, and creating policies that prioritize patient-centered care. Encouraging active involvement in healthcare decisions and promoting awareness about the benefits of PE can help bridge these gaps and improve healthcare outcomes in the region. Future research efforts must be directed toward the creation of standardized and validated measures of PE. This will facilitate the identification of predictors of PE and enable quantifying the effectiveness of strategies to enhance PE.
